# Correction: Viral escape-inspired framework for structure-guided dual bait protein biosensor design

**DOI:** 10.1371/journal.pcbi.1014398

**Published:** 2026-06-12

**Authors:** Yee Chuen Teoh, Mohammed Sakib Noor, Sina Aghakhani, Jack Girton, Guiping Hu, Ratul Chowdhury

There are errors in the Funding statement. The correct Funding statement is as follows: This work is supported in parts by the Iowa State University Startup Grant (Building a World of Difference Faculty Fellow), Center for Industrial Research and Service (CIRAS) Mini Grant, and NSF 22–599, Established Program to Stimulate Competitive Research (EPSCoR) RII Track-1, Award Number 2242763 to RC. MSN was funded by the NSF 22–599, Established Program to Stimulate Competitive Research (EPSCoR) RII Track-1, Award Number 2242763 grant. The funders had no role in study design, data collection and analysis, decision to publish, or preparation of the manuscript.
