# Supplementary figures and images for: Viral escape-inspired framework for structure-guided dual bait protein biosensor design
Source: PLoS Comput Biol. 2025 Apr 15;21(4):e1012964. doi: 10.1371/journal.pcbi.1012964 (PMC12021294; doi:10.1371/journal.pcbi.1012964)

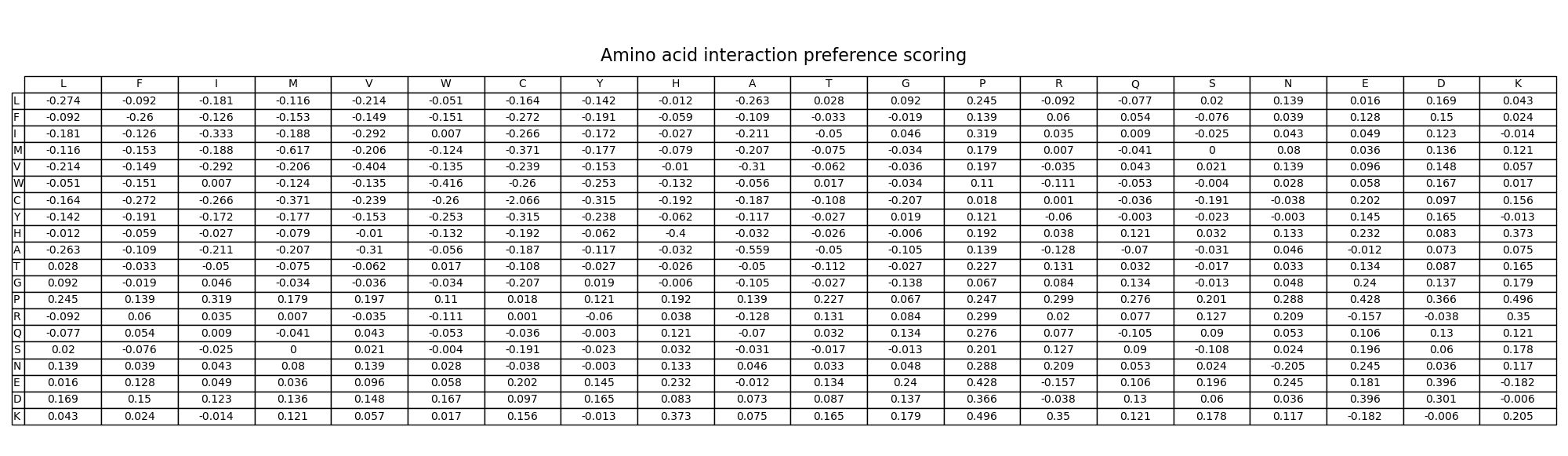

Supplement: S1 Fig — A more negative score represents strong attractive forces between two amino acids separated by optimal bonding distance (from Lennard-Jones potential; LJ 6–12 equation). The table is however biased away from Proline and towards Cysteine, i.e., pairwise preference scores for any amino acid with Pro are very high (positive) while those with Cys are very low (negative). This creates an imbalance in the integer optimization protocol introduced in CTRL-V-1 (see Methods). (TIFF) [file pcbi.1012964.s001.tiff]

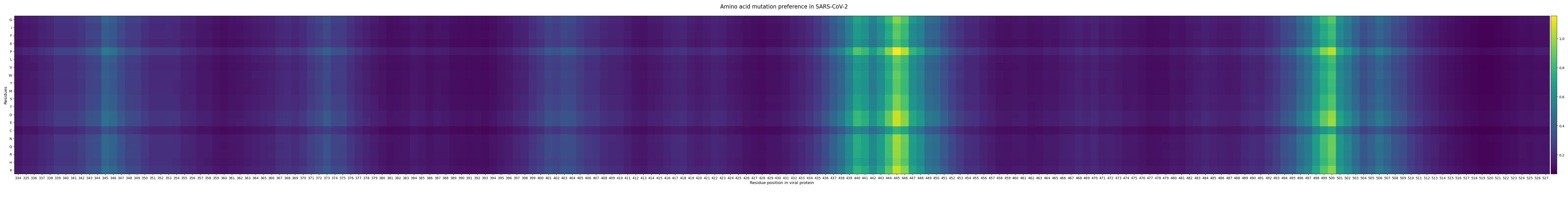

Supplement: S2 Fig — The bright vertical swatches represent the interacting residues from the spike protein (x-axis represents all amino acids on the viral spike RBD domain from N-terminus through C-terminus). The two horizontal streak likes - one bright (for Pro) and one dark (for Cys) indicates the super high and low preference for Pro and Cys during mutations on the spike protein. The choice is biased due to the imbalance in the pairwise amino acid preference score (S1 Fig). (TIFF) [file pcbi.1012964.s002.tiff]

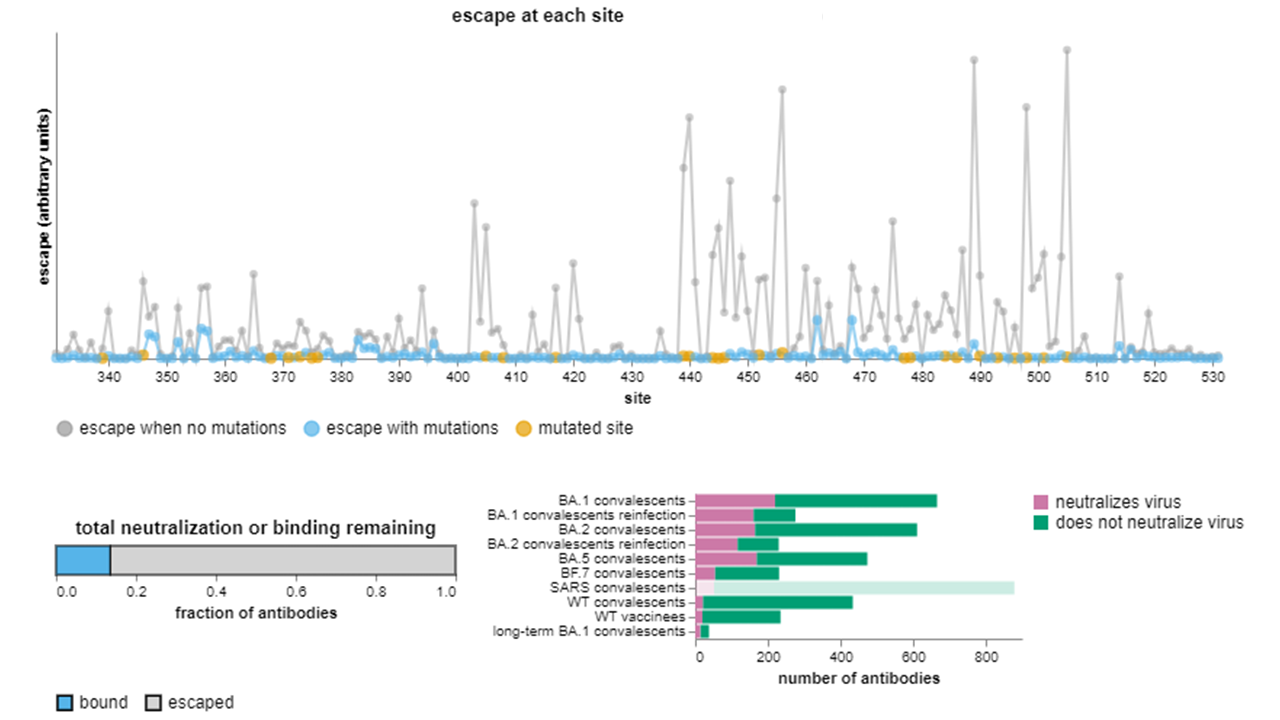

Supplement: S3 Fig — While CTRL-V-3 was only provided information about the Ly-CoV1404 neutralizing antibody to predict escape loci, the predictions recovered loci which, through mutations, aids the SARS-CoV-2 virus to escape from 36 reported neutralizing commercial antibodies. (TIFF) [file pcbi.1012964.s003.tiff]
