# Supplementary material for: Viral escape-inspired framework for structure-guided dual bait protein biosensor design
Source: PLoS Comput Biol. 2025 Apr 15;21(4):e1012964. doi: 10.1371/journal.pcbi.1012964 (PMC12021294; doi:10.1371/journal.pcbi.1012964)
Supplement: Attachment — Submitted filename: [Response to Reviewers] Teoh and Noor et al. 2025.docx [file pcbi.1012964.s008.docx]

# Reviewer 1

# Teoh *et al* have presented a manuscript that describes the mechanistic design of a high specificity biosensor protein. They describe use cases of 3 versions of differing versions of the program (CTRL-V) benchmarked against known experimental data on SARS-CoV-2 spike antigen, a monoclonal antibody, a human receptor and data from a DMS experiment and 30 circulating variants emerging from Spike. The manuscript covers the performance of the 3 iterations of the model in predicting true variants compared to false variants. CTRL-V-3 performs the best amongst the 3 when compared to these experimentally derived datasets and is beneficial in that it requires no training phase. I believe that this study is suitable for publication with the caveat, that this is not my exact field of expertise. The experimental design appears to be sound, although parts of the paper the relevance of the method should be clarified.

# We are happy to learn that the reviewer found this suitable for publication in PLoS Computational Biology. However, in response to the questions by other reviewers, we have improved the manuscript. We demonstrated the generalizability of CTRL-V on known experimental data on biosensors thereby validating our claim.

# Reviewer 2

We appreciate the reviewers’ questions our designed model and we address those point to point below.

## Question 1

*Is it safe to use the COVID-19 virus to construct a bio sensor (we hate the virus). Please justify this choice and state the measurement for guarantee the safety for the public.*

We understand and agree with the concern expressed by the reviewer. The objective of this work is to design a reliable computational framework to design dual-bait biosensors - which are molecules than can discriminately bind one protein/ peptide over another from a given pair of target proteins/ peptides. These computational models require a lot of experimental to understand the biochemical priors that drive the amino acid mutations to distinguish between two proteins. To this end, experimental datasets on a single system are very sparse and at best report sub-optimal improvements to the starting biosensor since the combinatorically explosive sequence space is almost impossible to sample experimentally. Here, SARS CoV-2's *in vivo* machinery acts as an analog computing machine to explore and identify the fittest sequence that accomplishes the dual-bait objective (i.e., binding to receptor, and avoiding binding to an antibody).

One fail-safe that guarantees that **CTRL-V cannot be used to design a viral variant of concern** is – for a variant to be virulent, the whole trimeric spike protein needs to be easily expressible on the viral capsid protein. We are isolating and only using the receptor binding domain (RBD) from one of the monomers of the spike - hence the model has no information viral expression mechanism. In addition, newer AI-based tools such as EVEscape and Evo-2 can generate future viral proteins and write whole genomes, respectively (even though Evo-2 puts a disclaimer that it has not been trained on viral genomes to predict newer ones, but the code is open-source). In light of these tools, CRTL-V is most detached from overall cellular machinery that drives viral evolution.

Overall - our key insight here is: the receptor binding domain (RBD) of viral spike proteins are a perfect exemplar of the dual-bait biosensor mechanism. RBD mutations allow it to escape antibody recognition while preserving receptor binding function. This dual selective pressure makes viral proteins particularly informative for studying protein-protein interaction specificity, a key aspect of biosensor design.

On the therapeutic side, CTRL-V can be leveraged to enhance public safety by helping to predict an ensemble of likely mutations to the viral RBD to drive putative design of broadly neutralizing peptides/ nanobodies/ antibodies. CTRL-V cannot pinpoint a single RBD variant as the possible next circulating variant, but an ensemble of sequences which provide insight to the structural biochemistry of the system and guide antibody design.

## Question 2

*Why the specific commercial LY-CoV1404 antibody is used, will other brand or other antibodies also fit in the method along with the framework?*

## We thank the reviewer for this query. Yes, any other antibody or multiple antibodies could be added into the framework. We chose to use the LY-CoV1404 because the experimental crystal structure of this antibody bound to the viral spike RBD (neutralization complex) was available. For any new antibody sequence, structure prediction using AlphaFold2 and docking with RBD is possible but would add uncertainties about the reliability of the structure of the complex. Additionally, since this antibody was administered in 2020, it is likely that every circulating variant of SARS-CoV-2 has some effect built into their sequences that helped them escape this antibody. This has been mentioned multiple times in literature and is also exemplified by the fact that additional boosting re-vaccination shots were required in following years.

## Question 3

*Why only hydrophobicity is considered important here? Are there any other indexes that is also important?*

While our results and discussion highlight hydrophobic interactions, CTRL-V actually considers multiple physicochemical properties that govern protein-protein interactions:

(1) Electrostatic Interactions: PyRosetta's energy function calculates Coulombic interactions between charged/polar residues, weighted by distance and environmental dielectric constant. This is exemplified in **Figure 7C** where N440K mutation disrupts a stabilizing interaction with Ser103 of the antibody due to charge repulsion, with the scoring term *fa_elec* capturing these electrostatic perturbations in our calculations.

Figure 7C caption reads: "V445H and G446L mutations together introduce a similar incompatible electrostatic surface through the insertion of polar groups close to hydrophobic ones and vice versa, respectively."

(2) Hydrophobic Effects: PyRosetta evaluates hydrophobic packing through the *fa_atr* (Lennard-Jones attractive) and *fa_rep* (Lennard-Jones repulsive) terms, while also considering solvation energy through the *fa_sol* term (https://docs.rosettacommons.org/demos/latest/tutorials/scoring/scoring). These terms help explain why mutations like F371 and P373 in KP.2 variant destabilize antibody binding by creating incompatible hydrophobic patches near polar antibody residues.

(3) Steric Considerations: Interface complementarity is evaluated through PyRosetta's interface_analyzer module, which calculates shape complementarity scores (*sc_value*) and buried surface area (https://docs.rosettacommons.org/docs/latest/application_documentation/analysis/interface-analyzer). This analysis revealed how N440K mutation's larger side chain (139.1Å² vs 80.8Å² surface area) disrupts the binding interface through steric clashes.

(4) Structural Stability: ProteinMPNN evaluates sequence-structure compatibility by learning from natural protein structures, through its deep learning framework. This helps eliminate mutations that might disrupt the RBD's fold, even if they appear favorable for antibody escape.

(5) Hydrogen Bonding Networks: PyRosetta's *hbond_sr_bb* and *hbond_lr_bb* terms evaluate backbone hydrogen bonds, while *hbond_sc* captures side chain hydrogen bonding patterns (https://docs.rosettacommons.org/docs/latest/rosetta_basics/scoring/score-types).

These terms were crucial in identifying how G446L mutation disrupts the original hydrogen bond network with antibody residue Ser97.

Our results may appear to emphasize hydrophobic effects because they were particularly relevant for the KP.2 escape mutations we analyzed, but the computational framework holistically considers all these physicochemical properties in predicting viral escape variants.

## Question 4

The presentation of the entire paper needs a major modification, please separate the discussion on biological termed analysis (for biologist) with the method explanation used like integer optimization formulation (for engineer), and simulation procedures (for programmer) as well.

We appreciate the reviewer's concern and have significantly rewritten the manuscript.

In addition, we have now added these following paragraphs in the updated Discussion section.

[*for biologists*](Lines 486-491): " CTRL-V offers a form technology for the mechanistic design of high-specificity dual bait biosensor proteins. We presented a sequential story for one of the use cases of CTRL-V on three versional implementations of CTRL-V benchmarked against known experimental data on SARS-CoV-2 spike RBD antigen (DM), commercial LY-CoV1404 antibody ($\text{TM}^{\text{×}}$), human ACE2 receptor (TM), deep mutational scanning data on the antigen, and 30 circulating variants that emerged from the spike antigen."

[*for engineers for optimization*] (Lines 498-519): " CTRL-V uses a tilted objective function that maximizes the difference between energy functions, predicting binary variables corresponding to amino acid choices at each position on the polypeptide backbone. This ensures the designed protein iteratively improves upon the current best solution to simultaneously enhance binding to one molecule while eliminating binding with another. The integer optimization model enables a global search of the sequence space with constraints that prevent revisiting previously explored solutions. Although CTRL-V-1, which utilizes integer optimization for sequence design and state-of-the-art tools for structure prediction and docking, yields the worst performance, we rationally discussed the reason for its poor performance. We also provide directions on how it could be improved in future versions and demonstrate it. CTRL-V-1 has an advantage in generating overall global optimal sequences *via* depth-first greedy search, but it is dependent on the amino acid preference score and is vulnerable owing to its dependency on – (a) the low efficacy of the latest structure prediction tools for viral proteins, (b) inability of docking tools to handle constrained multi-chain docking tasks (where only rotamer repacking needs to be done between two chains, and flexible docking needs to be done between the third chain and the common interface of all the three chains). CTRL-V-1 has the lowest recall rate and is GPU intensive due to the ESMfold step. CTRL-V-2 uses a more conventional sequence generation, structure updating, and docking protocols within PyRosetta, which has a moderate recall rate and is the least dependent on multiple tools cross-talking with each other. However, it has a long simulation time and only produces locally optimal sequences as it stochastically explores an umbrella of sequences at each step (breadth first). CTRL-V-3, with the addition of ProteinMPNN, performs best of the three and demonstrates efficacy in pinpointing select point mutations as escape variants. It has the same disadvantages as CTRL-V-2 and is GPU/CPU intensive due to the ProteinMPNN. "

[*for programmers*] (Lines 520-528): " CTRL-V is an open-source tool that integrates multiple off-the-shelf computational biology tools (ESMfold, HADDOCK3, PyRosetta, ProteinMPNN) with our novel integer optimization code in a single iterative framework. Users need to upload protein structure files (PDB format) for the paired protein complexes (antigen-antibody and antigen-receptor), with the final output delivered as a ranked list of point mutations in CSV format containing binding energy differences and structural metrics. The implementation is available through Google Colab for accessibility across computing environments. Our study was performed on modest hardware specifications (1 node with 8 processor cores, 64GB memory, NVIDIA A100 GPU), where we observed a non-linear increase in true-positive recovery rate with increasing generations of escape variants (**Figure 9**)."

## Question 5

*It looks the entire paper is comparing pro and con for the gradually improved platforms, from which, the reader can neither gain inside of the virus mutation escaping antibody, nor the inside of a potential flexible sensor design, or the appreciation of the accuracy of the Python model.*

We understand the sentiment behind the reviewer's statement and their attention to detail while reviewing our work.

While it is cursorily uninteresting if three progressively improving models are presented, we show, in detail, how workflows made up by stitching state-of-the-art tools for individual steps can be suboptimal overall. We provide analysis why an overall state-of-the-art computational tool might be fraught with lack of biological awareness when it comes to precision biotechnology such as biosensor design.

On the whole, CTRL-V involves steps including (a)structure evaluation, (b) protein-protein docking, (c) binding affinity assessment, and an (d) optimization framework to predict new sequences as a function of the prior three steps (a-c).

Now, there are more than a dozen computing tools for each of the steps (a), (b), and (c). For step (d) we contributed a novel integer optimization algorithm. So, we first made CTRL-V into a modular chassis, where the user can choose different combination of available tools for (a), (b), and (c).

Next, we wanted to show two exemplar combinations for tools for steps (a-c). The version 1 reflected the most light-weight, fast, deterministic (depth-first) implementation with the least computational overhead. The version 2 reflected the most compute intensive version combining all ML-based state-of-the-art tools for each step and including stochastic sequence design (which uses breadth-first search). We saw that both these implementations were non-performative and revealed the key bottlenecks of many of these state-of-the-art tools for applications as these. This was super informative to indicate the right strategy for version 3, which maximizes performance using reasonable compute resources (we have summarized it in **Figure 9**). The version 3 is shown to generalize for a non-viral case where it successfully identifies mutations to an experimentally validated residue in Raf kinase which helps it discriminate between two GTPase enzymes (Ras and Rap1a).

The progressive nature of the study serves a dual purpose - while version 3 achieves the highest accuracy, the documented limitations of versions 1 and 2 provide the scientific community with valuable insights into potential pitfalls in protein-protein interaction modeling, effectively serving as an exemplar of a negative workflow that can guide future workflow building.

Our systematic comparison across versions revealed that accurate prediction requires: (a) consideration of structural stability alongside binding effects, (b) evaluation of multiple physicochemical properties rather than just interaction energies, and (c) incorporation of sequence-structure compatibility assessment. These principles extend beyond viral proteins to general protein-protein interaction prediction.

## Question 6

*Please explain the importance of electrostatic contact, how it is compared with hydrophobicity?*

Electrostatic contacts and hydrophobic interactions play complementary but distinct roles in protein-protein binding. In some cases, electrostatics guide molecules into proximity (long-range effects), while hydrophobicity stabilizes the complex through short-range interactions. Conversely, electrostatic repulsion can reduce hydrophobic binding affinity if charged residues disrupt hydrophobic clustering.

In our study electrostatic contacts determine specificity through charge-based interactions as seen in V445H and G446L mutations where together this introduces a similar incompatible electrostatic surface through the insertion of polar groups close to hydrophobic ones and vice versa, respectively. The distance-dependent nature of these interactions (Coulomb's law) means they can influence binding even at longer ranges than hydrophobic effects. Hydrophobic interactions, while shorter-range, provide essential contributions to binding strength through van der Waals forces and the hydrophobic effect. This is demonstrated by the F371 and P373 mutations in KP.2 SARS CoV-2 variant, which creates a hydrophobic patch that destabilizes binding with the antibody's hydrophilic surface. CTRL-V evaluates both interaction types using PyRosetta's energy scoring function, with *fa_elec* terms capturing electrostatics and *fa_atr/fa_rep* terms handling hydrophobic packing.

## Question 7

*Why there are a number of 0% in the simulation table, is it caused by the particular virus sequence or computer code/model design?*

The 0% values in Table 1 stem from (a) Cysteine and proline biases in the amino-acid interaction scoring system (from Jha *et al.*) used in CTRL-V version 1, and (b) ML-based structure predictor ESMFold's inability to predict structures of viral proteins in CTRL-V version 2, not from the viral sequence itself. ESMFold's training set does not have enough viral structures in their training set since very few viral proteins and their variants have been crystallized.

For version 1, the 0% precision/recall was seen because the scoring system used was heavily biased towards selecting proline and not selecting cysteine mutations due to the amino acid preference scoring matrix (as shown in Figure 4B). This limitation highlights why we chose to present version 1 as a negative computational study. The integer optimization formulation is not flawed since we have prior work showing how similar architectures can be leveraged to design proteins.

Two of the many works by us which use integer optimization to design proteins, and show agreement with experiments:

1. https://www.nature.com/articles/s41467-018-06097-1

2. https://pubmed.ncbi.nlm.nih.gov/31430120/

For these studies, we had used the classical physics-based force-fields like CHARMM and GROMACS to score protein interactions. We have also shown that these force-fields can be used to score SARS-CoV-2 spike with ACE2 receptor (1) and designed (experimentally unverified) antibody (2) interactions in our prior work

1. https://pubmed.ncbi.nlm.nih.gov/32983400/

2. https://pubmed.ncbi.nlm.nih.gov/36111441/

Here, we wanted to explore the utility of a different scoring system just for version-1. In version-2 we fix this and use classical force field through the PyRosetta, to score the interactions.

However, for version 2, the 0% recovery occurred because PyRosetta's stochastic sequence design initially focused on mutations that appeared energetically favorable but were structurally unstable. This revealed the need for incorporating sequence-structure compatibility checks, which we subsequently addressed in version 3 using machine-learning based ProteinMPNN, leading to significant improvements in performance across the commercial antibodies included in this study.

# Reviewer 3

## Question 1

Highlight Novelty: Emphasize how CTRL-V differs from existing escape-prediction and biosensor design frameworks. Include quantitative comparisons to establish its advantage.

We thank the reviewer for the valuable suggestion. Additional portions have been added to the end of the *Introduction* in the revised manuscript.

"… Recent advances in computational biology have led to various approaches for predicting protein-protein interactions and viral mutations, yet each address only partial aspects of the complex challenge of viral escape prediction. Machine learning approaches have led recent developments, with notable advances such as the language model-based approach by Hie et al. innovatively applies natural language processing to viral evolution, achieving over 85% prediction accuracy for SARS-CoV-2 mutations by treating viral sequences as linguistic constructs. Similarly, EVEscape combines deep learning on historical viral sequences with biophysical information to predict mutation fitness effects. While these approaches excel at capturing evolutionary patterns, they fundamentally rely on historical sequence data, potentially limiting their ability to predict unprecedented evolutionary paths. Also, they cannot be applied more broadly as a protein engineering platform to design dual-bait proteins that discriminate between different interaction partners. Both are specifically tailored for viral mutation prediction only and cannot provide any structural underpinning of how mutations allow any protein to discriminate between different interaction partners.

Sequence-based interaction tools like MaSIF and DeepPPISP focus specifically on protein-protein interaction prediction. MaSIF employs geometric deep learning for surface pattern recognition, while DeepPPISP predicts interaction sites using sequence information alone. However, these tools are not trained on viral proteins given the low number of experimentally crystallized structures exists for both (a) viral protein and host receptor, and (b)) viral protein and neutralizing antibody. This is why such tools are poised/ trained to capture the dynamic nature of viral evolution or the simultaneous interaction with multiple proteins.

More specialized tools have recently emerged for antibody-antigen interactions. mCSM-AB2 uses graph-based machine learning to predict antibody-antigen binding affinity changes, but it:

- assumes static complex structures and do not account for energy minimization of the complex,
- cannot account for the interaction between antigen and receptor to capture escape-related biochemical objective - and hence not poised to predict escape variants

PRIEST employs deep learning on temporal sequence data to predict mutations but:

- focuses solely on evolutionary patterns of the sequence - with no information about structure or direct selection pressure imposed by specific host antibodies and host receptor proteins.

A multi-task learning approach by Gross and Sharan et al. (2022) represents a step forward by analyzing experimental escape data from multiple antibodies simultaneously but remains specifically focused on antibody escape in SARS-CoV-2's receptor binding domain without addressing receptor binding. The lack of structural data provides no interpretability in terms of the energetics of binding.

CTRL-V provides a structural insight too individual residue-level contribution of energetics hence provides a strong basis for understanding why and how proteins of interest can discriminate between different interaction partners (metal ions, nucleotides, and small molecules). In addition, CTRL-V distinguishes itself from these existing approaches through several key innovations.

1. It uniquely addresses the problem of (viral) protein A's evolution by simultaneously evaluating both (antibody escape) unbinding to protein X and (receptor) binding to protein Y. This better reflects the actual selective pressures on viral mutations, as demonstrated by its successful identification of escape mutations in the KP.2 variant.
2. Unlike tools dependent on historical data or experimental training sets, CTRL-V employs a modular, physics-based framework that integrates established computational tools to analyze protein-protein interactions without requiring prior training data.
3. Furthermore, CTRL-V's modular architecture allows it to function as a general dual-bait biosensor design platform that can investigate how mutations allow any protein to discriminate between different interaction partners, setting it apart from virus-specific tools while providing detailed structural and biophysical understanding of mutation mechanisms.

## Question 2

## Address Scalability: Discuss computational resource requirements and how CTRL-V can be scaled or optimized for broader adoption.

We appreciate the reviewer's interest in understanding hardware prospecting for CTRL-V.

We have now elaborated our setup for the study and addressed potential optimization based on resources. Kindly refer to the updated discussion session.

"… Our benchmarking was performed on modest hardware specifications (1 node with 8 processor cores, 64GB memory, NVIDIA A100 GPU), where we observed a non-linear increase in true-positive recovery rate with increasing generations of escape variants (**Figure 9**). A more improved CTRL-V with a longer duration can potentially recover more single-point mutations that appear for a given antigen (DM)-antibody (TM)-receptor (non-TM) biosensor triad (**Figure 9**; see **SI File 3**).

The framework's modular architecture distributes computational load across three key steps, each can be optimized for specific hardware resources. PyRosetta's structure refinement employs gradient-based minimization that benefits from CPU thread-level parallelism. The sequence design step using ProteinMPNN can be optimized through GPU acceleration which demonstrated near-linear scaling on GPU clusters . These specifications represent minimum requirements for effective implementation, with performance expected to improve on more powerful computing infrastructure. Thus, this platform can be optimized for broader applications."

## Question 3

Improve Validation:

Strengthen computational validation by comparing CTRL-V outputs with experimental datasets from other viral escape studies or biosensor applications. Consider including wet-lab validation to support computational predictions.

We agree with the reviewer's view that a computational prediction should be validated experimentally to add credibility.

To this end, we would like to point that in the original manuscript we benchmarked CTRL-V with mutations that appeared during the pandemic in 30 major mutations, spanning 39 variants recognized by WHO. While wet-lab validation of the platform for designing protein biosensors is part of our future endeavor – we have now included the generalizability of CRTL-V (version 3) for successfully predicting experimentally known mutations and key mutational loci to Raf kinase that promotes differential binding to two GTPases: Ras and Rap1a. We have added this to a new sub-section in the Results of the updated manuscript.

## Question 4

Expand Generalizability: Provide examples or mock results to demonstrate CTRL-V's potential applications beyond SARS-CoV-2.

Following our answer from the previous question, we have explored a naturally occurring signaling system involving Raf kinase and its differential binding to the GTPases Ras and Rap1a using CTRL-V version 3. Kindly refer to the “*Validation of CTRL-V using the Raf-Ras-Rap1a discriminatory system”* under the Results section.

"To further validate the CTRL-V platform's ability to predict mutations that enable proteins to discriminate between different binding partners, we explored the well-characterized interaction system involving Raf kinase and its differential binding to the GTPases Ras and Rap1a inspired by the study by Serebriiskii et al. Raf kinase serves as a critical node in the MAPK signaling pathway, where its ability to selectively respond to Ras but not Rap1a (despite their 56% sequence identity) plays a central role in signal transduction specificity. The molecular basis for this discrimination has been extensively characterized. While both Ras and Rap1a interact with Raf's Ras-Binding Domain (RBD) and Cysteine-Rich Domain (CRD), key differences in binding affinity and conformational effects determine signaling outcomes. This system represents an ideal test case for CTRL-V (version 3), as it exemplifies the naturally occurring phenomenon where a protein (Raf) discriminates between structurally similar partners (Ras and Rap1a), preferentially binding and responding to one over the other. Using crystal structures of the Ras-Raf complex (PDB: 4G0N) and the Rap1a-Raf complex (PDB: 1C1Y), we applied CTRL-V version 3 to predict mutations in the Raf RBD that would further enhance its discrimination between Ras and Rap1a. CTRL-V successfully identified mutations in Raf's RBD that enhanced its binding specificity toward Ras. Specifically, mutations at the interface residues 69E and 66D were predicted to increase the binding energy differential between Ras and Rap1a interactions (Figure 8). The 69E mutation replaces valine and establishes stronger electrostatic interactions with Ras compared to Rap1a, while the 84E mutation demonstrates how charge distribution affects binding interface complementarity."

## Question 5

Enhance Methodological Clarity: Provide detailed descriptions of integer programming, PyRosetta, and ProteinMPNN steps. Ensure all methods are reproducible by detailing parameter choices and workflow configurations.

We agree with the reviewer on the importance on reproducibility of method/ results.

We have now added a code-availability statement

To this end, the whole CTRL-V code is made available through our GitHub (linked in the manuscript), so that there is no ambiguity/ need to code it up from scratch. We also make CTRL-V usable easily as a web tool on Google Colab notebook.

**"Code availability**

We have made CTRL-V available as a freely accessible tool as a Google Colab

(https://colab.research.google.com/drive/1Pkw8MNW4uGnqp5wfW6AIyfM1nORsOyRE?usp=sharing), and the standalone program downloadable from GitHub (https://github.com/ChowdhuryRatul/CTRL-V). "

## Question 6

Focus on Presentation:

Use clearer, high-resolution images for structural results and create more descriptive legends.

Reorganize tables to facilitate comparisons across CTRL-V versions.

We appreciate the reviewer's emphasis on visual clarity in our manuscript. Regarding the image quality, we have carefully prepared all structural figures at high resolution (minimum 300 dpi) using PyMOL's ray-tracing functionality to ensure maximum clarity of molecular details. All structural figures feature consistent coloring schemes and viewing angles to facilitate comparisons across variants.

For the tables, we specifically designed Table 1 to allow direct comparison across all three CTRL-V versions, with performance metrics (accuracy, precision, recall) organized to enable immediate assessment of each version's relative effectiveness.
